# Mechanical behaviors of hydrate-bearing sediment with different cementation spatial distributions at microscales

**DOI:** 10.1016/j.isci.2021.102448

**Published:** 2021-04-16

**Authors:** Yanghui Li, Peng Wu, Xiang Sun, Weiguo Liu, Yongchen Song

**Affiliations:** 1 Key Laboratory of Ocean Energy Utilization and Energy Conservation of Ministry of Education, Dalian University of Technology, Dalian, 116024, P. R. China

**Keywords:** Sediment Geochemistry, Mechanical Property, Microstructure, Porous Material

## Abstract

Unlike the conceptual models, the natural hydrate spatial distribution in sediments is multitype and presents different coalescence degrees. In this study, we present pore-scale triaxial test results for hydrate-bearing sediments with different hydrate spatial distributions for the first time. It shows that the specimen with a more dispersed hydrate distribution yields later and exhibits larger peak strength. Correspondingly, the localized deformation develops more slowly, and the shear band is steeper and thinner. The cementation failure in the specimen with a more dispersed hydrate distribution develops more slowly. However, the changing rate of the pore space characteristic does not seem to be affected by the hydrate spatial distribution. Moreover, the specimen with a more dispersed hydrate distribution has a larger hydrate-sand interfacial area, and further axial loading would increase it rapidly firstly, and then the increasing rate would be slowed down since the cementation structure failure.

## Highlights

• Dispersed hydrate spatial distribution strengthens the sediment more significantly
• Dispersed hydrate spatial distribution steepens and thins the shear band
• Hydrate distribution seems to not affect the pore space evolution during shearing
• The cementation interfacial area evolution was calculated during shearing

## Graphical abstract

**Figure:**
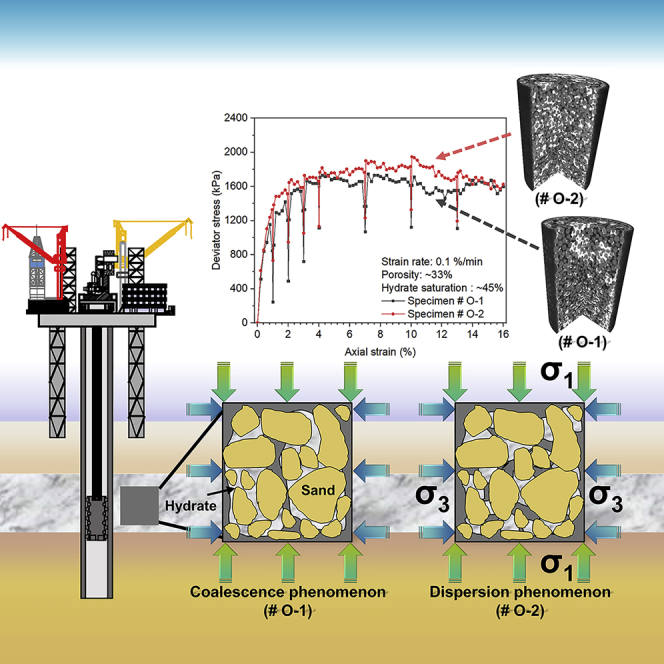

## Introduction

Natural gas hydrates (NGHs) are crystalline cage-like compounds formed by the interaction of methane and other hydrocarbon gases with water (Englezos, 1993). The formation and stability of NGHs requires a relatively high pressure and low temperature; therefore, NGHs are typically found in submarine continental margins, permafrost, and Arctic regions (Lu, 2015). Since the 1980s, the United States (Francisca et al., 2005), China (Song et al., 2014; Zhou et al., 2014), Japan (Yun et al., 2011), India (Lee and Collett, 2009), South Korea (Lee et al., 2013), and other countries have implemented specific investigation and development plans for studying NGHs. To date, NGHs have been found in more than 150 locations worldwide, and their carbon reserves are estimated to be 1.8×10^3^Gt, which is approximately twice the global carbon resource of traditional fossil energy (Boswell and Collett, 2011). NGHs are desired to replace traditional fossil energy and become a clean new energy source in the future (Liu et al., 2020; Wu et al., 2020), and the main fluid-saturated environment can be classified into two categories: the first is the excess water environment, which exists in the most marine environment; and the second is the excess gas environment, which is mainly found in the gas-rich system where the gas recycles into an upward-migrating base of hydrate stability (Hyodo et al., 2013a). However, the hydrate reservoir existing in the excess gas is thought to have greater development potential since the free gas can be jointly exploited for increasing the natural gas production (Yoneda et al., 2019). Generally, the current development of NGHs is in an important stage of progressing from field trials to commercial exploitation (Wang et al., 2021; Zhou et al., 2018).

Geomechanical issues related to hydrate-bearing sediments are currently the biggest problem restricting the development of NGHs. Unlike conventional oil and natural gas resources, NGHs existing in the sediment are mainly in a form of cementation or skeleton support (Clennell et al., 1999; Lei and Seol, 2018). The frictional heat generation from drilling tools, the salty variation in the drilling mud, and the well pressure changes could lead to cementation failure and undesirable disturbances in the hydrate-bearing sediment (Yoneda et al., 2019), which could result in a degradation in their geomechanical characteristics. This in turn could trigger a series of geohazards, such as wellhead instability, stratum deformation, submarine landslides, and even climate change (Ding et al., 2019; Jiang et al., 2015).

Geomechanical research on hydrate-bearing sediments has commenced since the 1970s, and the initial focus was on interpreting seismic reflection profiles according to the small-strain elastic wave velocity (Stoll et al., 1971). Later, along with the discovery of highly permeable hydrate-bearing sediments with a high hydrate saturation, researchers started considering NGHs as a potential resource (Francisca et al., 2005; Lee and Holder, 2001; Tsuji et al., 2004). A large number of geomechanical experiments have been conducted on hydrate-bearing cores and remolded hydrate-bearing specimens from the laboratory and field to evaluate their geomechanical characteristics, and they are influenced by many factors, such as the hydrate saturation (Hyodo et al., 2013b, 2014), effective confining pressure (Choi et al., 2018; Hyodo et al., 2013a), fines content (Lee et al., 2010) and other factors (Iwai et al., 2018; Li et al., 2019b). Detailed literature reviews about geomechanical research on hydrate-bearing sediments have been comprehensively discussed elsewhere (Priest and Hayley, 2019; Yan et al., 2020). Nevertheless, the vast majority of studies did not consider the hydrate cementation spatial distribution influence on the mechanical behavior of hydrate-bearing sediments due to the lack of an effective nondestructive technique. Moreover, in nature, the hydrate cementation pore habit was reported to be heterogeneous affected by the effective stress, pore-size-dependent capillary pressure, and hydrocarbon accumulation history, which has been assessed directly by X-ray computed tomography (CT) (Lei et al., 2019; Le et al., 2020) or magnetic resonance imaging (MRI) (Ji et al., 2020; Song et al., 2015; Zhang et al., 2020b) and indirectly by comparing the measured seismic velocities and those calculated via elasticity models (Dvorkin et al., 2000). Luo et al. (2015) studied the effect of a heterogeneous distribution of the hydrates on the wave velocity of the sediment and found that the compressional wave velocity increases with the hydrate saturation, while the heterogeneous distribution of the hydrates can have a remarkable influence on the results, especially in the low-saturation condition. This indicates that the heterogeneous spatial distribution of the hydrate will have an impact on the small-strain elastic characteristics. Shen et al. (2016) and Shen and Jiang (2016) compared the influence of the hydrate occurrence on the mechanical behavior of hydrate-bearing sediments using conceptual models of the cementing type and grain coating type with discrete element method (DEM) simulations, and the impacts on the stress-strain response were discussed. Triaxial tests on hydrate-bearing interlayered sediments were also conducted, and it was found that the stress-strain response of hydrate-bearing interlayered sediments is substantially different from that of hydrate-bearing sediments, and mechanical parameters such as the strength, elastic modulus, and initial yielding modulus are deeply affected by the hydrate distribution (Dong et al., 2019; Li et al., 2018), illustrating that the hydrate spatial distribution should be taken into consideration when evaluating the geomechanical behavior of hydrate-bearing sediments. With the introduction of X-ray CT, Wu et al. (2020a) studied the failure behavior of hydrate-free zones, cementing hydrate-bearing zones and patchy cluster hydrate-bearing zones in the different stages of the stress-strain curve.

As illustrated above, studies on the influence of the hydrate cementation spatial distribution on the mechanical behavior of hydrate-bearing sediments are quite rare. In this study, by the microfocus X-ray CT-based gas hydrate triaxial testing apparatus (Li et al., 2019a), we present hydrate spatial distribution influence on the mechanical behavior of hydrate-bearing sediments at pore-scale. In addition to the traditional stress-strain responses, we further explore the evolution of the localized deformation, hydrate particle number and size. Moreover, pore network modeling (PNM) is also used to describe the pore space evolution during triaxial shearing for the first time. A simple algorithm for calculating the hydrate-sand interfacial area is also proposed, and the impact of the hydrate cementation spatial distribution on the interfacial area during shearing is also discussed.

## Results

### The spatial distribution influence of hydrates on the pore system

**Figure 1:**
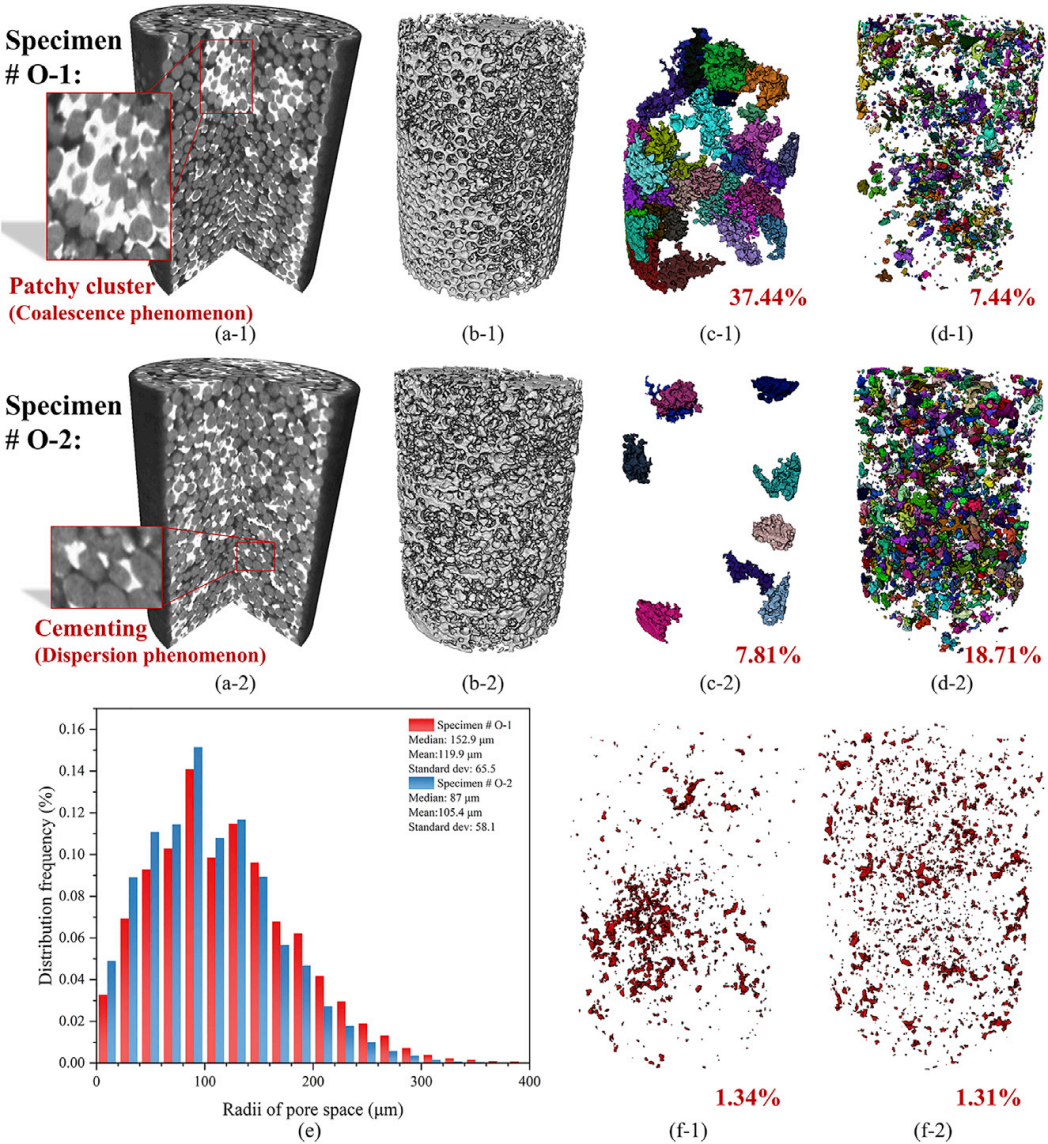
The vertical-sectional view, hydrate occurrences, and pore space radii distribution of hydrate-bearing sediments #O-1 and #O-2 (A) vertical-sectional view of the specimens; (B) the hydrate in the specimens; (C) the hydrate particles which are larger than 10 mm^3^ in the specimens; (D) the hydrate particles which are smaller than 1 mm^3^ in the specimens; (E) the pore space radii distribution; (F) the unconnected pore space in specimens.

**Figure 2:**
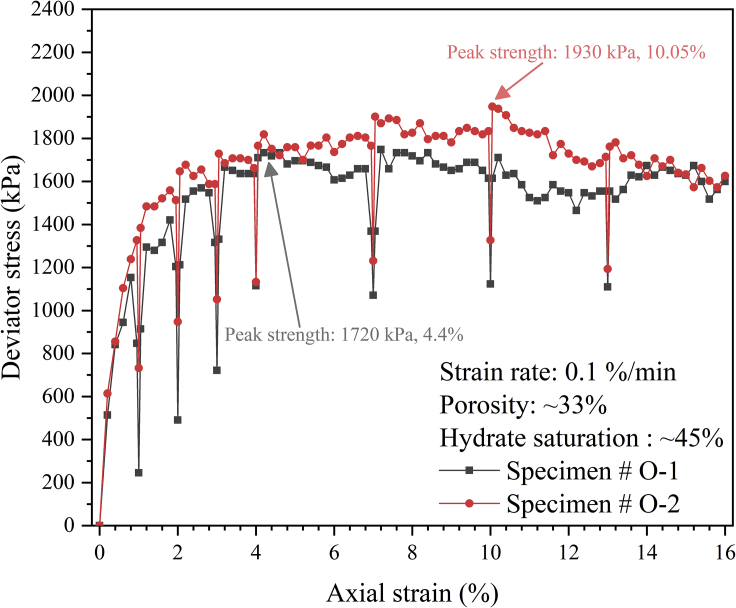
The stress-strain relationship of the specimen with different hydrate spatial distributions

With the microfocus X-ray CT-based gas hydrate triaxial testing apparatus (Li et al., 2019a), the CT image sequence of the hydrate-bearing sediment at different axial strains were obtained. Figures1A-1 and 1A-2 show the vertical-sectional views of specimens #O-1 and #O-2, and the hydrate cementation has also been rendered in Figures1B-1 and 1B-2, respectively. The spatial distributions of the hydrate cementations are quite different due to the different specimen preparation processes. To better show the spatial concentration of the hydrate cementation, the hydrate particles are labeled and sieved according to their sizes. Figures1C-1 and 1C-2 show hydrate particles with sizes larger than 10 mm^3^ in the two specimens, accounting for 37.44% and 7.81%, respectively, while Figures1D-1 and 1D-2 show hydrate particles with sizes smaller than 1 mm^3^ in the two specimens, accounting for 7.44% and 18.71%, respectively.

For the specimen #O-1, the hydrate presents a large-scale coalescence phenomenon which wraps multiple sand particles, reaching a hydrate saturation of 100% locally. And this occurrence is also called a “patchy cluster”, which has been found in previous studies (Lei and Seol, 2020; Yoneda et al., 2016). For the specimen #O-2, the hydrate presents a dispersed distribution phenomenon, and more hydrate cementations occur in the menisci between the sand particles or encapsulated on the sand particle surface. This occurrence is also called “cementing”, which is quite common in the excess gas environment, and can substantially stiffen and strengthen the sediment (Lei et al., 2019). In addition, the “pore-filling” hydrate occurrence was not found for both specimens as the same as Lei and Seol (2020) and Yoneda et al. (2016). This is because although water can migrate among the sand particles (Le et al., 2020; Nikitin et al., 2020), it is still physically impossible to float at the pore space in a gas excess environment. Figure 1E shows the hydrate spatial distribution influence on the radii distribution of the pore space. For the specimen #O-1, it was found that the specimen in which the hydrate presented a large-scale coalescence phenomenon showed larger radii of the pore space. This is because although the hydrate clusters completely occupy the pore space in some local areas of the specimen #O-1, in the other areas the imbalance of the hydrate distribution would result in a significant volume of pore space without hydrates, whose radius is quite large. Therefore, the overall pore space radius distribution is larger for the specimen in which hydrates coalesce more heavily. In addition, it has to be noted that although the median radii of the pore space in the two specimens are around 100 μm, the hydrate particle could be even larger than 10 mm^3^. This is because the hydrate particle is not an isolated small particle only existing in a single pore as the idealized conceptual model (Clennell et al., 1999; Wu et al., 2020c), but a cluster existing in multiple pores and throats at the same time.

The determining of the unconnected pore space was achieved by the module “axis connectivity” in Avizo 9.0.1 following Kong et al. (2018). Figures1F-1 and 1F-2 show the unconnected pore space distribution vertically for the two specimens, and they occupy 1.34% and 1.31% of the total pore space, respectively. This illustrates that for the unconnected pore space the hydrate spatial distribution has little influence on its volume ratio but has a relatively large influence on the spatial position. The specimen in which hydrates present a dispersed distribution has a more homogeneous unconnected pore space distribution, and this could affect the production capacity during exploitation to some extent.

### Stress-strain relationship and corresponding localized deformation development

Figure 2 shows the hydrate spatial distribution influence on the stress-strain relationships for the two specimens, and the deformation processes can also be found in Videos S1 and S2 respectively. Also, terminologically, the axial strain refers to the ratio between the sediment changed length and the corresponding original length, and the deviator stress refers to the difference between the major principal stress and minor principal stress (Wu et al., 2021).

**Figure 3:**
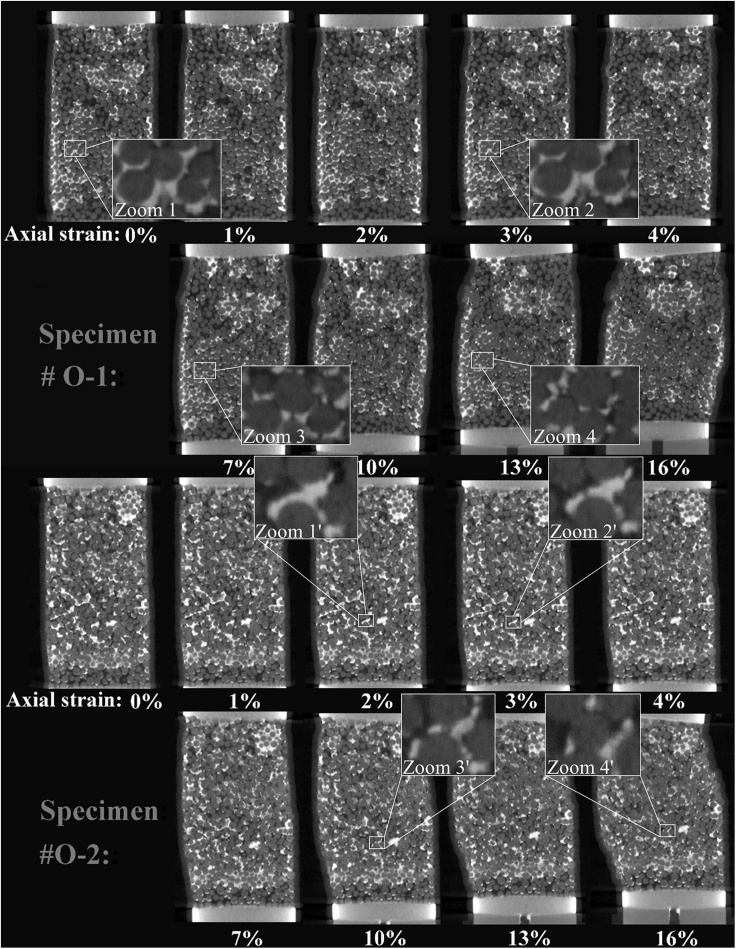
Longitudinal cross-sectional CT images of the specimens during shearing process

VideoS1. The deformation process of the specimen #O-1,related to the figures 2 and 3

VideoS2. The deformation process of the specimen #O-2,related to the figures 2 and 3

There are some drops in the curves; for example, the stress relaxation at an axial strain of 1%, which is due to the necessary stop of axial loading during the CT scans. This phenomenon has been widely reported (Cheng and Wang, 2019; Cheng et al., 2020), and no obvious mechanical influence was found.

In this study, both specimens show a slight strain-softening phenomenon, which is consistent with the triaxial test results for remolded hydrate-bearing sandy specimens (Dong et al., 2019, 2020), which is due to the failure of hydrate cementation under high stress (Priest and Hayley, 2019).

It is worth noting that the peak strength of the specimen #O-1 (1720 kPa at an axial strain of 4.4%) is smaller than that of the specimen #O-2 (1930 kPa at an axial strain of 10.05%), and the specimen #O-1 yields earlier than the specimen # O-2, which illustrates that the specimen in which hydrate particles present a large-scale coalescence phenomenon would show a weaker resistance to deformation, macroscopically showing a quicker failure and a lower peak strength. Similar results were also found by Masui et al. (2005) using different hydrate-bearing specimen remolding techniques. It was found that the remolding techniques could determine the hydrate cementation distribution between sand particles, and under similar hydrate saturation the hydrate-bearing specimen with weaker cementation effect present lower peak strength. Besides, the difference would be reduced with the increasing hydrate saturation. This is because the strength difference of the two mainly comes from the area without cementation, but with the increase of saturation, the area without cementation will gradually decrease and thus, the strength difference between them will also be further reduced.

However, the initial slopes of the stress-strain curves for both specimens are almost the same, illustrating that hydrate spatial distribution hardly affects the initial elastic properties of the sediment. This is because the pore space with very weak or no support of hydrate cementation is the main deformation area during the initial shearing process (Wu et al., 2020b), and for the two specimens with the same hydrate saturation and porosity, the volume of such pore space could be almost the same; thus, the initial elastic properties are similar.

**Figure 4:**
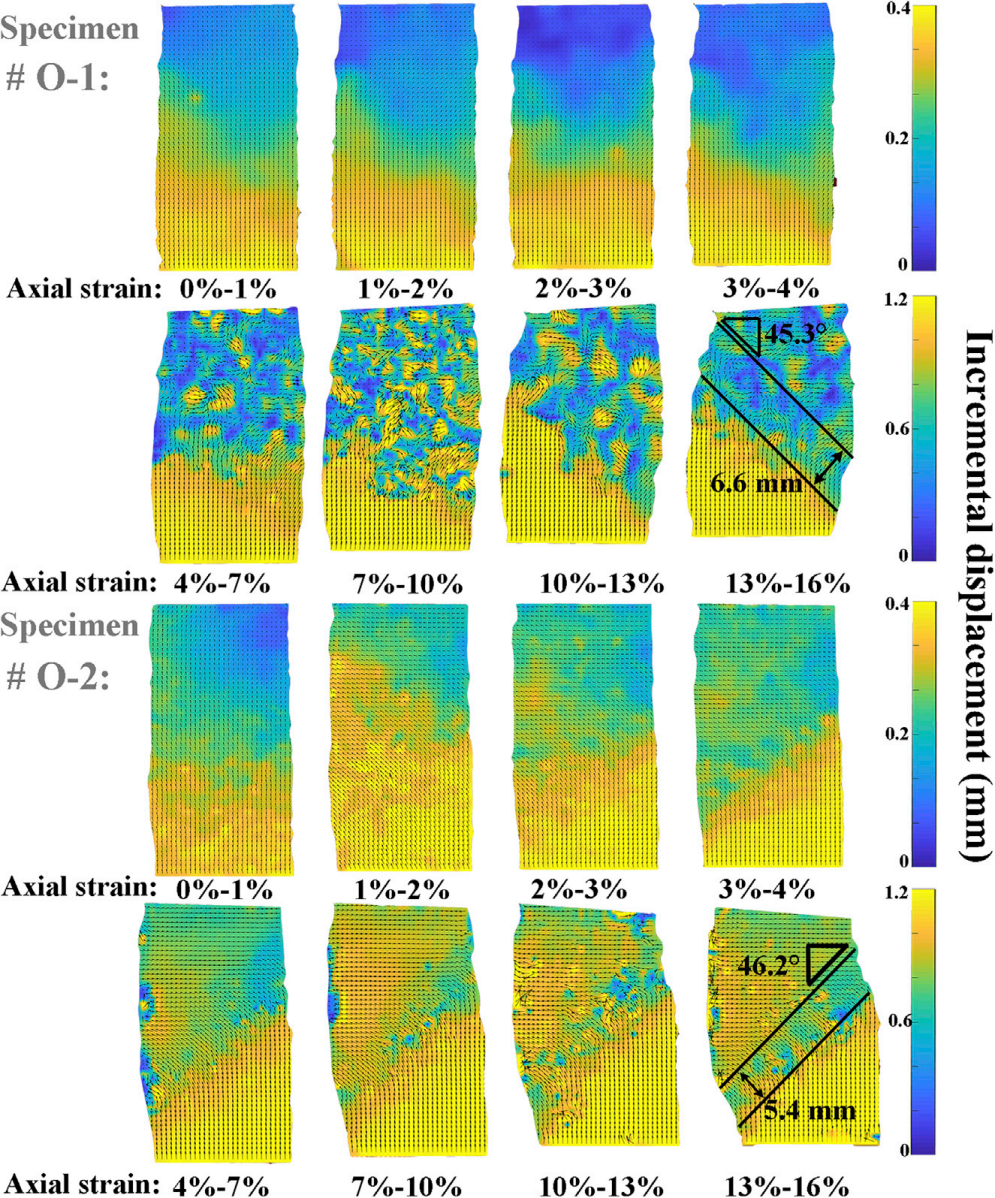
Corresponding localized deformations of the specimens during shearing process

Figures 3 and 4 show the longitudinal cross-sectional CT images for the two specimens during the shearing process and the corresponding localized deformation. The localized deformation was determined using the particle image velocimetry (PIV) technique (Thielicke and Stamhuis, 2014), which is achieved by tracking a small area in the reference and deformed images to identify the incremental displacement. This has also been widely used to visualize the localized deformation (or shear band) development (Yoneda et al., 2013, 2016; Kato et al., 2016). The vector expresses the tracked path between each deformation process, while the color bar shows the scalar value of the vector. The shear band is a slip zone with different directions of movement on both sides that usually runs through the specimen obliquely. It is also a macroscopic manifestation of strain localization in the process of soil catastrophes, and it is the symptom and prerequisite of most rock and soil damage. Thus, when there is an inconsistent movement direction on both sides of an oblique region in a PIV diagram, the transition zone in the middle is usually considered a shear band (Yoneda et al., 2016). However, when the original objects in the reference area changed significantly, this technique would lose its effectiveness locally since it cannot find the corresponding area in the deformed images.

Figure 4 shows that localized deformation starts as a broad zone before the peak strength and then progressively become thinner during continued loading. Finally, the shear band becomes concentrated with a determined inclination angle and thickness. This is consistent with previous studies on hydrate-bearing sediments with plane strain shear tests (Thielicke and Stamhuis, 2014; Kato et al., 2016; Kajiyama et al., 2016) and CT-based triaxial shear tests (Wu et al., 2020a; Yoneda et al., 2016).

Meanwhile, the localized deformation in the specimen #O-1 mainly occurs when the axial strain increases from 0% to 1%, while for the specimen #O-2, it mainly occurs when the axial strain increases from 3% to 4%, illustrating that the localized deformation in the specimen in which hydrates present a dispersed distribution develops more slowly. This is mainly because when the hydrate saturation remains constant, the more heavily the hydrate coalesces, the more areas exist with non/low local hydrate saturation, which are prone to deform due to the lack of support. Thus, due to “Cannikin’s law”, the localized deformation in specimens in which hydrates present a large-scale coalescence phenomenon develops more quickly. According to the Mohr-Coulomb theory (Coulomb, 1776), the inclination angle of the shear band is proportional to the internal friction angle of the specimen, and the formula is expressed as follows:

(Equation 1) $$\theta =\frac{\pi }{4}+\frac{\phi }{2}$$

where *θ* is the inclination angle of the shear band and *ϕ* is the internal friction angle.

The inclination angle of the shear band determines the development direction of rock and soil failure, which plays an important role in accurately reproducing the progressive failure process of rock and soil. From Figure 4, it can found that the shear band in the specimen #O-2 (46.2°) are steeper than that of the specimen #O-1 (45.3°), and the shear band in the specimen #O-2 (5.4 mm, corresponding to 4.6 times the average particle diameter of the matrix) are thinner than that of the specimen #O-1 (6.6 mm, corresponding to 5.6 times the average particle diameter of the matrix), illustrating that the shear band is steeper and thinner in the specimen in which hydrates present a dispersed distribution with a larger internal friction angle.

From Figure 4, many spots showing high incremental displacement can be observed in specimens #O-1 and #O-2, especially when the axial strains are larger than 4% and 10%, respectively. This is mainly due to two points: on the one hand, in case the hydrate saturation remains constant, the patchy clusters consume larger amounts of hydrates according to Figures1C-1 and 1C-2; thus, there are more non/weak hydrate cemented areas around the patchy clusters in the specimen, making it easier for the cluster to migrate to these directions. On the other hand, the fast cementation failure is also an important reason for this phenomenon, the crushing and shedding behavior of the hydrates will prevent the PIV technique from tracking to the previous area in the CT images.

### Analysis of the hydrate particles and pore space characteristics

**Figure 5:**
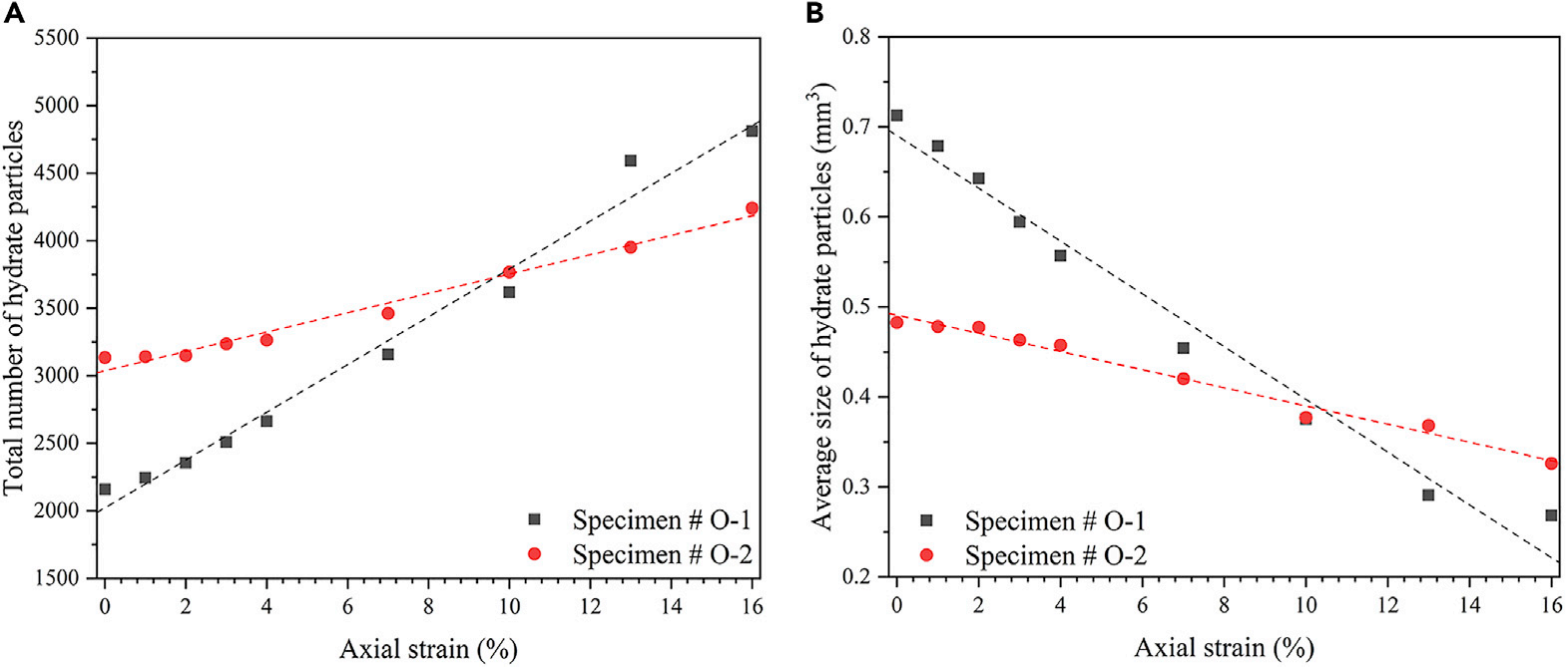
The variation hydrate particle parameters for the specimen with different hydrate spatial distributions (A) the total number; (B) and average size.

Figures5Aand 5B show the influence of the hydrate spatial distribution on the total number and the average size of the hydrate particles during the shearing process. Also, the hydrate particle sizes were obtained according to the cumulative size of the voxels consisting of each hydrate particle; specifically, it was achieved by the module “label analysis” in Avizo 9.0.1. When the axial strain is 0%, the total number of hydrate particles in specimen #O-1 is less than that of specimen #O-2, while the average size of hydrate particles in specimen #O-1 is larger than that of specimen #O-2, mainly because the hydrate in specimen#O-1 presents a more obvious coalescence phenomenon.

With continuous loading, the hydrate particles will be broken into more and smaller pieces due to the grinding effect between the particles and the tension failure since the local expansion, which can also be found in zooms 1, 1′, 2 and 2′ of Figure 3. Therefore, the average size of the hydrate particles will decrease while the number of hydrate particles increases correspondingly. However, the slope of the variation is smaller in specimen #O-2, illustrating that the cementation failure process develops slower in the specimen whose hydrates present a dispersed distribution. This may be because for the specimen whose hydrates present a dispersed distribution, the hydrate particles sticking to the sand particles can be smaller generally, and these sand particles can rotate and rearrange more easily. This flexible behavior of the hydrate particles can avoid the hydrate particles being compressed and broken among the sand particles to some extent. In addition, macro-statistically, the fracture and crushing of the large hydrate cluster in the specimen #O-1 can bring about more substantial decrease and increase in the average size and the total number, respectively.

**Figure 6:**
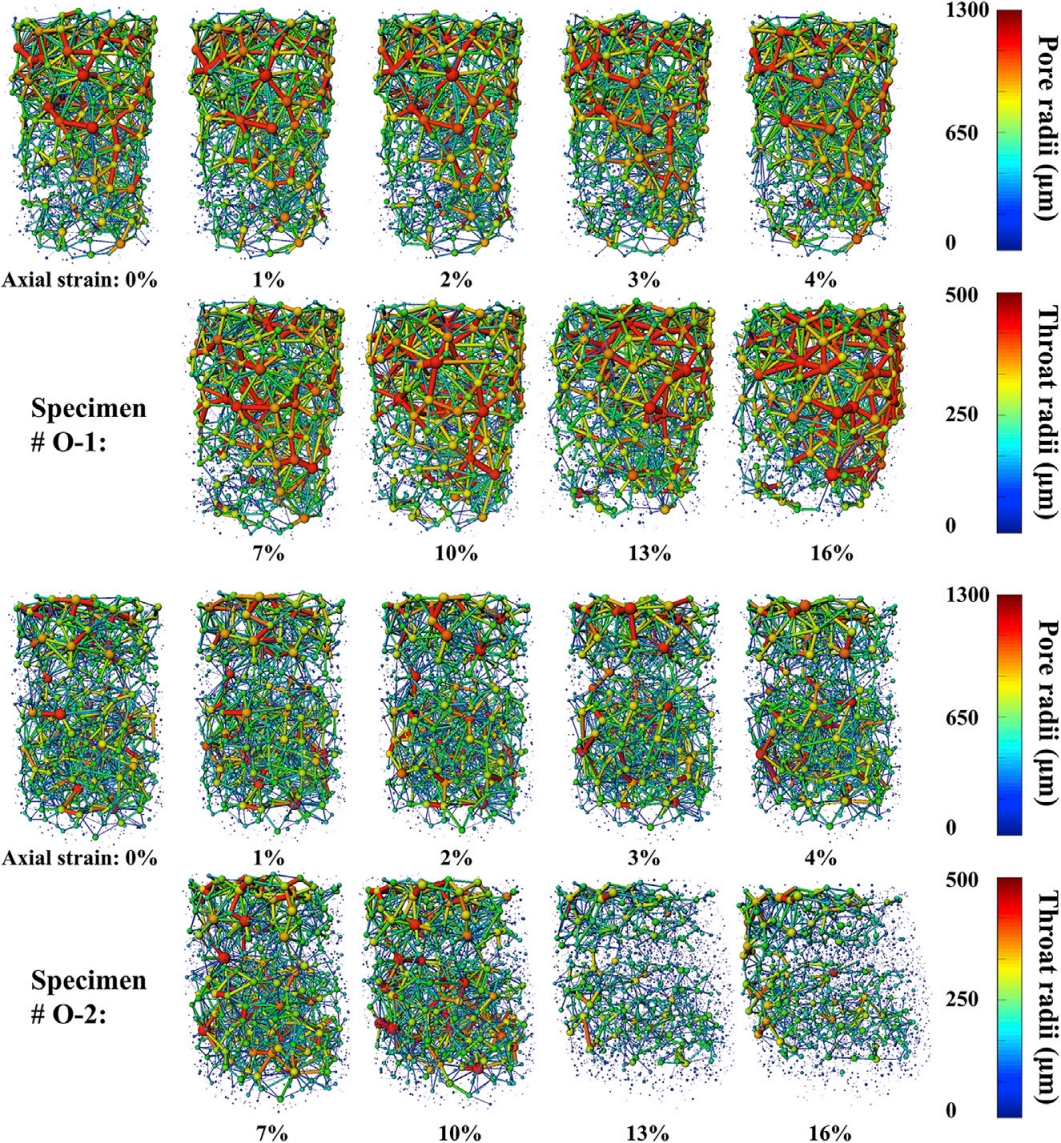
The PNM model evolution with different hydrate spatial distributions during shearing process

**Figure 7:**
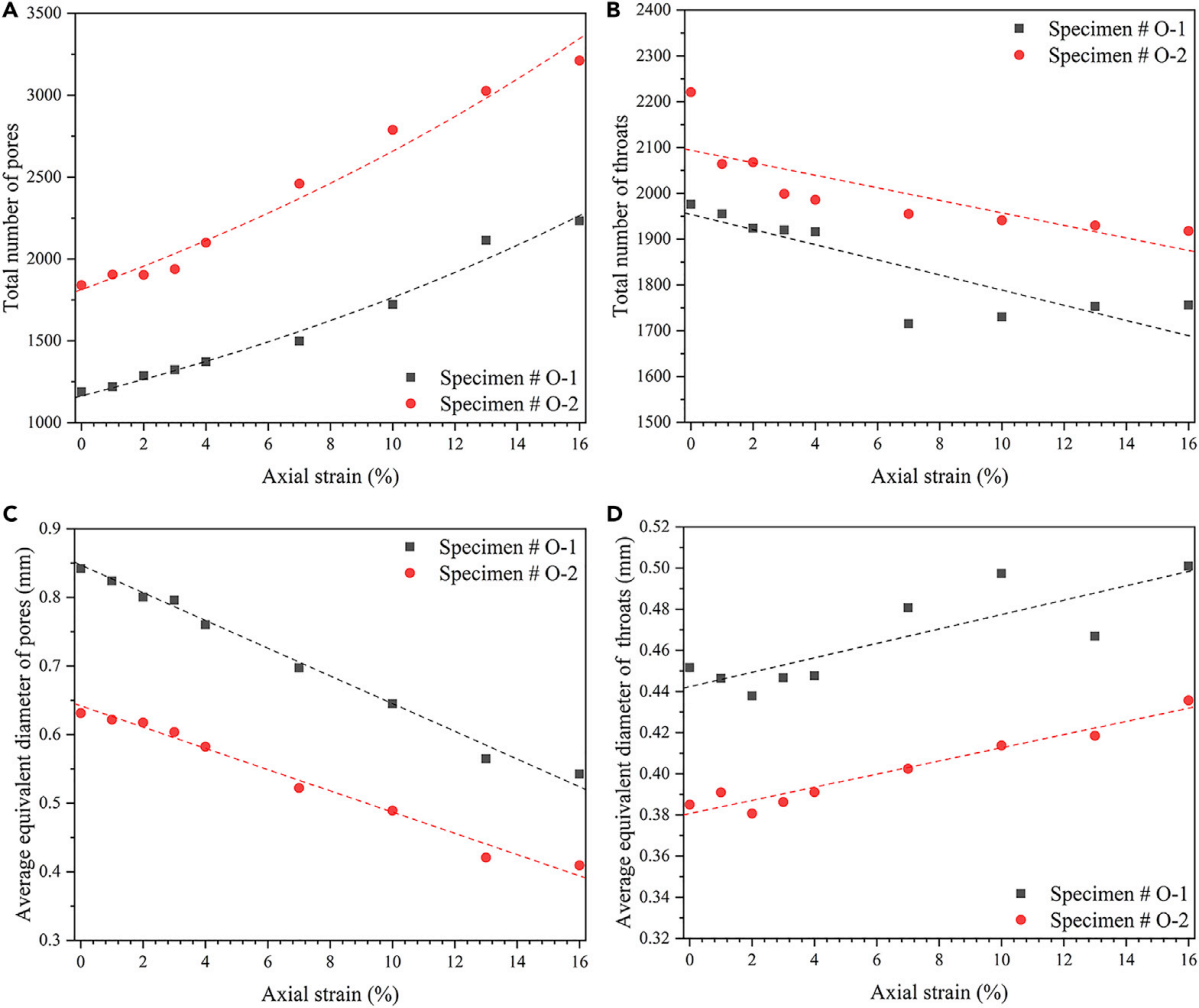
The parameter development of PNM with different hydrate spatial distributions during shearing process (A) evolution in the total number of pores; (B) evolution in the total number of throats; (C) evolution in the average equivalent diameter of pores; (D) evolution in the average equivalent diameter of throats.

The PNM was used to further study the hydrate spatial distribution influence on the pore space evolution, and the maximal ball method proposed by Dong and Blunt (2009) was used to extract the pore network, and its feasibility has been validated in previous studies (Wang et al., 2020; Zhang et al., 2020a). The basic principle of PNM is to make the largest inscribed ball in each pore space formed by voxels. Usually, the largest ball is regarded as a pore, and the smallest ball in the middle of the two balls is regarded as a throat. The PNM models with different axial strains are rendered in Figure 6, in which the sphere corresponds to the pore and the rod corresponds to the throat; however, it is difficult to visually observe the evolution, and the index parameters are summarized in Figures7A–7D.

Figures7A–7D present the total number and the average equivalent diameter evolution of the pore and the throat for the two specimens. Based on the values at the axial strain 0%, for the specimen in which hydrates present a large-scale coalescence phenomenon (#O-1), the total number of the pore and the throat is smaller while the average equivalent diameter is larger. This phenomenon confirms the result about the pore space radii distribution in the section “The Spatial Distribution Influence of Hydrates on the Pore System”, which is because the imbalance of the hydrate distribution could lead to a significant volume of the large pore space without hydrates. In addition, for the pore space where is no hydrate around, the complexity is not high; thus, the pore and throat would be considered to be larger and fewer during the judgment of PNM (Zhang et al., 2020a).

With continuous loading, for the pores of both specimens, the total number increases while the average equivalent diameter decreases, and the parameters vary in the opposite direction for the throat. This may be because the particle rotation and rearrangement will compress and encroach upon the pore, and when the particles come close enough, the original large pore could be divided into multiple smaller pores, which would lead to an increase in the total number and a decrease in the average size of the pore. During the shearing process, as shown in zooms 2-4 and 2′-4′ of Figure 3D, the hydrate particles sandwiched between the sand particles will be compressed and broken quickly due to the stress concentration, and these trapped hydrate particles will be grounded and transferred to the adjacent pores; therefore, the throat will be dredged, and the total number will decrease while the equivalent diameter will statistically increase. Moreover, it should be noted that there is a tendency for the mentioned throat parameter variation to slow along with continued loading, which may be due to the limit of the transport effect caused by the rotation of the sand particles (Cheng and Wang, 2018a). In addition, the slopes of the curves in Figures7A–7D are basically the same, which illustrates that the hydrate spatial distribution has little effect on the pore space evolution during the shearing process. The factor of the host material (grain size distribution, sand particle shape, etc.) could have a more pronounced influence (Kajiyama et al., 2017; Wang et al., 2015).

### Analysis of the interfacial area between the hydrate particles and sand particles

**Figure 8:**
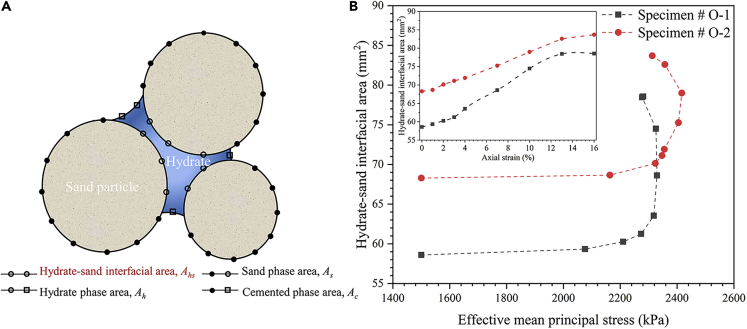
The hydrate-sand interfacial area evolution with different hydrate spatial distributions during shearing process (A) illustration of the hydrate-sand interfacial area algorithm; (B) the evolution of the hydrate-sand interfacial area.

As shown in Figure 8A, a simple algorithm for calculating the hydrate-sand interfacial area was proposed for evaluating the failure behavior of hydrate cementation, and the algorithm is as follows:

(Equation 2) $$A_{hs}=\frac{A_{s}+A_{h}-2\times A_{c}}{2}$$

Where *A*_*hs*_ is the hydrate-sand interfacial area, *A*_*s*_ is the sand phase area, *A*_*h*_ is the hydrate phase area and *A*_*c*_ is the cemented phase area. Specificity, the sand phase area *A*_*s*_ and the hydrate phase area *A*_*h*_ are calculated based on the binarized image sequence of sand phase and hydrate phase by the module “Area3d” in Avizo 9.0.1 directly. This module could statistics the surface area for each specific phase according to the surface area of outside voxel bodies representing corresponding phase. In order to calculate the cemented phase area *A*_*c*_, an addition process for the binarized image sequences of sand phase and hydrate phase was conducted first, and then the module “Area3d” was employed to calculate the cemented phase area *A*_*c*_.

Figure 8B presents the evolution of the hydrate-sand interfacial area with different hydrate spatial distributions along with the effective mean principal stress and the axial strain. For Figure 8B, it can be found that the initial hydrate-sand interfacial area of specimen # O-1 is smaller than that of specimen #O-2, and this is because the larger the size of the hydrate particle, the smaller will be the specific surface area.

Initially, within the effective mean principal stress of 2200 kPa, the hydrate-sand interfacial areas for both specimens hardly change with the increase in the effective mean principal stress. This corresponds to an axial strain of 2%, in which a uniform deformation is found according to Figure 4, and the pore space structures where there is a hydrate-free or low hydrate concentration reported to be the main deformed object (Wu et al., 2020b; Miyazaki et al., 2011). Therefore, the main change could occur in the sand-sand interfacial areas instead of the hydrate-sand interfacial areas with the initial increase in the effective mean principal stress.

After that, when the effective mean principal stress is greater than 2200 kPa, which corresponds to the yield stress for the two specimens in Figure 2, the hydrate-sand interfacial area increases rapidly and even straightens. This is mainly because from the process in zooms 1-3 and 1′-3′ of Figure 3, the continuous loading enhances the extrusion between the sand particles and hydrate particles, and the hydrate-sand interfacial area increases rapidly. When the extrusion reaches a certain degree, it is difficult for the particles to undergo further extrusion until cementation failure occurs. Also, as shown in zooms 3, 3′, 4 and 4′ of Figure 3, when the hydrate particles sandwiched between the sand particles have been grounded into multiple smaller pieces with larger specific surface areas, since the hydrate particles still stick and are sandwiched between the sand particles instead of being transferred to the pore completely, the hydrate-sand interfacial area increases directly.

Finally, further loading will lead to a complete failure of the hydrate-cemented structure. In turn, the increase in the hydrate-sand interfacial area slows down, and the corresponding effective mean principal stress even decreases slightly due to the decrease in the bearing capacity of the sediment.

## Discussion

Unlike the idealized models, the natural hydrate spatial distribution in sediments is multitype and presents different coalescence degrees. In this study, two specimens with the same saturation and porosity but different hydrate coalescence degrees were remolded to investigate the hydrate spatial distribution influence on the mechanical behavior of hydrate-bearing sediment, and the main conclusions are summarized as follows:

1 The hydrate spatial distribution seems to have no influence on the initial elastic properties of the specimen, and both specimens exhibit strain-softening. However, the specimen in which hydrates present a dispersed distribution yields later and shows a larger peak strength.
2. The localized deformation in the specimen in which hydrates present a dispersed distribution develops slower, and the shear band is steeper and thinner, illustrating a larger internal friction.
3. The specimen in which hydrates present a dispersed distribution has more multiple and smaller hydrate particles, and the cementation failure develops more slowly.
4. The specimen in which hydrates present a dispersed distribution has more multiple and smaller pores. With increasing axial loading, the total number of pores will increase and the average equivalent diameter will decrease correspondingly, while the index parameter evolution in the throats shows the opposite phenomenon. In addition, the hydrate spatial distribution has little impact on the changing rate of these index parameters.
5. The specimen in which hydrates present a dispersed distribution has a larger hydrate-sand interfacial area. Along with the axial loading, the hydrate-sand interfacial area will increase rapidly and even straighten. Further loading will lead to the complete failure of the hydrate-cemented structure, which would lead to the slowing of the increase in the hydrate-sand interfacial area.

### Limitations of the study

Compared with the previously mentioned studies, the strain-softening phenomenon in this study is not so significant, and this could be mainly induced by the coarse round host material: The spherical sand particles would cause much less cementing contacts in comparison with the natural random-shape sand particles, which would weaken the cementing influence on the mechanical property significantly. Besides, the little specific surface area caused by the coarse grain size distribution is also an important factor, which would decrease cementing contacts significantly from the point of view of the whole specimen. In addition, because of the spherical shape, some rearrangement behavior of the host sediment particles, such as rotation and crossing over, could be hard to achieve due to the lack of an effective fulcrum, which would reduce the strain-softening phenomenon greatly.

It should be noted that the hydrate cementing area is greater than or equal to the interfacial area between the hydrate particles and the sand particles. In addition, the proposed algorithm is only used for evaluating the interfacial area between the hydrate particles and the sand particles.

## STAR★Methods

### Key resources table

**Table:** 

REAGENT or RESOURCE	SOURCE	IDENTIFIER
**Chemicals, peptides, and recombinant proteins**		

Xenon (99.99%)	DSG	N/A

**Software and algorithms**		

InspeXio	Shimadzu	https://www.shimadzu.com
ImageJ2	(Schindelin et al., 2017)	https://imagej.nih.gov/ij
Avizo 9.0.1	Thermo Fisher Scientific	https://www.thermofisher.com
PIVlab	(Thielicke and Stamhuis, 2014)	https://www.mathworks.com/matlabcentral/fileexchange/27659-pivlab-particle-image-velocimetry-piv-tool-with-gui
MATLAB2017a	MathWorks	https://www.mathworks.com/products/matlab.html

**Other**		

Microfocus X-ray CT system	Shimadzu	SMX225CTS-SV
The data related to the pore space radii distribution, stress-strain relationship, hydrate particle number and average size evolution, PNM parameter evolution and hydrate-sand interfacial area evolution.	This paper	https://doi.org/10.5281/zenodo.4646784

### Resource availability

#### Lead contact

Further information and requests for resources and reagents should be directed to and will be fulfilled by the lead contact, Peng Wu (pengwu_1118@outlook.com).

#### Material availability

This study did not generate new unique reagents.

#### Data and code availability

All data produced or analyzed for this study are included in this paper and its supplementary information files.

### Method details

#### Specimen preparation

To eliminate the random particle shape impact on the mechanical properties and in consideration of the CT resolution for particle image velocimetry (PIV) analysis (Thielicke and Stamhuis, 2014), spherical quartz glass sand (BZ-1, Asone Co., Japan) was chosen as the host material. The particle size ranged from 0.991 to 1.397 mm, the average particle diameter *D*_*50*_ was 1.19 mm, and the density *G*_*s*_ was 2.5 g/cm^3^.

Xenon gas is used to generate xenon hydrate (XeH) instead of methane gas to enhance the phase contrast in the CT images. Moreover, it must be noted that the physical characteristics between the XeH and NGH are also quite close (Chaouachi et al., 2015; Ning et al., 2012), as are the pore-scale occurrences between XeH and NGH following the similar formation procedure (Lei et al., 2019; Le et al., 2020). Furthermore, the substitutability of XeH for NGH for some pore-habit evolution studies has been widely accepted (Chaouachi et al., 2015; Yang et al., 2016).

The specimen preparation process consists of host specimen preparation and an *in situ* hydrate generation process, and the specifics are as follows (Wu et al., 2020b):

First, deionized water (1.6 g) was frozen into ice and compacted into pieces by a tamper. For the specimen with larger hydrate cementation particles (specimen #O-1), the compacting was conducted two times, while for the specimen with smaller hydrate cementation particles (specimen #O-2), the compacting was conducted ten times. However, since there was only 1.6 g of ice, the screening process would cause great loss; thus, the specific particle size distribution of the ice was not measured.

Then, the frozen dry quartz glass sand (18.6 g) was mixed with the grounded ice particles. Then, the mixed sand was compacted into five layers in a rubber membrane with a mold at −4°C.

After that, the host specimen was frozen at −4°C for convenient installation, the specimens #O-1 and #O-2 were measured to be ϕ 19.2×38.4 mm and ϕ 19.3×38.6 mm respectively.

Then, metal permeable stones were installed at both ends of the frozen host specimen, after which the frozen host specimen was placed on the pedestal of the triaxial testing apparatus.

After the assembly of the triaxial testing apparatus, the confining pressure and pore pressure were increased to 1.6 MPa and 0.6 MPa by nitrogen gas and xenon gas to make the host specimen stand by itself and achieve consolidation. Meanwhile, the temperature was set at 18°C to melt the ice for 10 minutes.

Finally, the hydrate generation was conducted by cooling the confining fluid temperature from 18°C to 7°C (note that the XeH stability required 13.3°C at 0.6 MPa). During the whole process, the pore pressure of the xenon gas was kept constant at 0.6 MPa by the plunger pump. XeH was believed to be fully generated when xenon gas consumption stopped, which took about 30 hours. According to the consumed gas volume and the XeH molecular formula of Xe·6.04H₂O, the conversion ratios of the water were 96.8% and 97.9% for specimens #O-1 and #O-2, respectively.

After the specimen preparation, consolidated drained shear tests were conducted with an effective confining pressure of 1.0 MPa and a strain rate of 0.1%/min.

#### X-ray CT test and postprocessing

Two types of X-ray CT scans with different distances from the X-ray source to the object (SOD) were performed: One was the full CT scan with an SOD of 270 mm, and the entire specimen could be scanned. Another was the partial CT scan with an SOD of 180 mm for higher magnification, and only the central specimen could be scanned. For the partial CT scan, the vertical height of the scanned region was manually increased after axial loading to ensure that the scanned region was always the center of the specimen, and the value was one-half of the axial displacement. That is, when the axial strain was 0%, the slice number was 936, and the corresponding height was 28.08 mm. When the axial strain was 10%, the slice number was 843, and the corresponding height was 25.27 mm. The axial displacement was stopped during CT scanning to avoid ghosting and blurring (Cheng and Wang, 2018a, 2018b). After CT scanning, axial loading was started again at the same strain rate, and the specimens were fully and partially scanned at axial strains of 0%, 1%, 2%, 3%, 4%, 7%, 10%, 13%, and 16%. All the CT scans were performed at 200 kV and 50 μA with an image size of 1024×1024 voxels, and the voxel sizes were 44 μm/voxel (SOD=270 mm) and 30 μm/voxel (SOD=180 mm) respectively.

**Figure 9:**
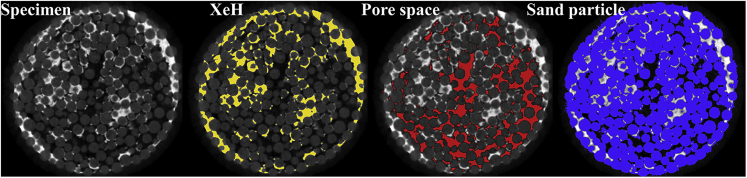
The segmented effect for the components

For postprocessing, the 2D CT images were reconstructed into a 3D volumetric model using InspeXio, and noise removal was conducted with a 3D median filter using ImageJ2. PIV analysis was conducted using MATLAB, and quantitative analysis and volume rendering were conducted using Avizo 9.0.1. Moreover, the image segmentation method was conducted mainly according to the gray value distribution of the image sequences, and the components in the specimen are divided into the pore space, the sand particles, and the XeH. In addition, the pore space refers to the pore space without XeH. For the CT images from the full CT scan, the confining fluid, rubber membrane, and metal permeable stones have to be removed using a mask first, and then the thresholds of the different components (the pore space, the sand particles, and the XeH) were determined by repeated comparisons of the volumes of the different components obtained from the CT images to the real volumes (Wu et al., 2020a). The specific segmented effect could be found in Figure 9, and each phase could be reflected effectively. The real specimen volumes were calculated by the size dimensions, which were 11.1 mm^3^ and 11.3 mm^3^ for specimens #O-1 and #O-2, and the real sand particle volumes were calculated by dividing the mass with density, which were both 7.44 mm^3^. Furthermore, the real XeH volumes were calculated by the consumed xenon gas, which were 1.72 mm^3^ and 1.74 mm^3^ for specimens #O-1 and #O-2. While the real pore space volumes were obtained by subtracting the real volumes of sand particle and XeH from that of specimen, which were 1.94 mm^3^ and 2.12 mm^3^ for specimens #O-1 and #O-2. In addition, the final hydrate saturation and porosity (ratio of the pore space volume without XeH to the whole specimen volume) were calculated at 46.7% and 33.1% for specimen #O-1 and 45.41% and 33.9% for specimen #O-2 from the CT images, which errors are no more than 1%. For the CT images from the partial CT scan, the confining fluid and rubber membrane were also removed using a mask first. However, it has to be noted that the gray value thresholds of the different components were different from the CT images obtained from the full CT scan. Thus, in this study, the component thresholds in the partial CT scan were determined according to the distribution ratio of the existing gray value in the CT images obtained from the full CT scan.

## Supplementalinformation

Table S1. Datasets of pore space radii distributions, related to the figure 1E

Table S2. Datasets of the stress-strain relationships,related to the figure 2

Table S3. Datasets of the hydrate particle number and average size evolution,related to the figure 5

Table S4. Datasets of PNM parameter evolution,related to the figure 7

Table S5. Datasets of the hydrate-sand interfacial area evolution,related to the figure 8

## Footnotes

Supplemental information can be found online at https://doi.org/10.1016/j.isci.2021.102448.
